# Treatment of node-positive endometrial cancer: chemotherapy, radiation, immunotherapy, and targeted therapy

**DOI:** 10.1007/s11864-023-01169-x

**Published:** 2024-01-04

**Authors:** Elizabeth A. Tubridy, Neil K. Taunk, Emily M. Ko

**Affiliations:** 1 Division of Gynecologic Oncology, Department of Obstetrics & Gynecology, Perelman School of Medicine at the University of Pennsylvania, Philadelphia, PA 19104 USA; 2 Department of Radiation Oncology, Perelman Center for Advanced Medicine, Philadelphia, PA 19104 USA; 3 Leonard Davis Institute of Health Economics, University of Pennsylvania Health Systems, Philadelphia, PA USA; 4 Penn Center for Cancer Care Innovation, Abramson Cancer Center, University of Pennsylvania Health Systems, Philadelphia, PA USA

**Keywords:** Nodal metastases, Endometrial cancer, Chemotherapy, Radiation, Immunotherapy, Targeted therapy

## Abstract

The standard of treatment for node-positive endometrial cancer (FIGO Stage IIIC) in North America has been systemic therapy with or without additional external beam radiation therapy (RT) given as pelvic or extended field RT. However, this treatment paradigm is rapidly evolving with improvements in systemic chemotherapy, the emergence of targeted therapies, and improved molecular characterization of these tumors. The biggest question facing providers regarding management of stage IIIC endometrial cancer at this time is: what is the best management strategy to use with regard to combinations of cytotoxic chemotherapy, immunotherapy, other targeted therapeutics, and radiation that will maximize clinical benefit and minimize toxicities for the best patient outcomes? While clinicians await the results of ongoing clinical trials regarding combined immunotherapy/RT as well as management based on molecular classification, we must make decisions regarding the best treatment combinations for our patients. Based on the available literature, we are offering stage IIIC patients without measurable disease postoperatively both adjuvant chemotherapy and IMRT with carboplatin, paclitaxel, and with or without pembrolizumab/dostarlimab as primary adjuvant therapy. Patients with measurable disease post operatively, high risk histologies, or stage IV disease receive chemoimmunotherapy, and vaginal brachytherapy is added for those with uterine risk factors for vaginal recurrence. In the setting of endometrioid EC recurrence more than 6 months after treatment, patients with pelvic nodal and vaginal recurrence are offered IMRT and brachytherapy without chemotherapy. For measurable recurrence not suitable for pelvic radiation alone, chemoimmunotherapy is preferred as standard of care.

## Introduction

Endometrial carcinoma (EC) is the most common gynecologic malignancy diagnosed in the USA [1]. While the majority of EC is diagnosed at early stage, 8% have spread to pelvic or para-aortic lymph nodes at the time of diagnosis [2]. The 2009 FIGO Staging system defines node positive EC to be stage IIIC1 (pelvic nodal involvement alone) or stage IIIC2 (para-aortic involvement with or without pelvic lymph node involvement) [3]. Risk factors for lymph node metastasis include increased depth of myometrial invasion (DOI), Grade 2 or 3 histology, lymphovascular invasion (LVSI), and non-endometrioid histology [4]. In patients with apparent uterine confined disease, the NCCN considers a minimally invasive surgery (MIS) approach to be standard. Randomized clinical trials such as LAP2 have indicated the feasibility of MIS staging and improved post operative quality of life in clinical stage I to IIA uterine cancer patients [5]. Surgical staging for EC requires lymph node assessment. Several phase III randomized control trials including the SENTI-ENDO Trial [6], the FIRES trial [7], and the SENTOR trial [2] have assessed the feasibility of sentinel lymph node biopsy (SLNB) rather than a full pelvic and para-aortic lymphadenectomy. Currently NCCN guidelines prefer SLNB as opposed to lymphadenectomy in patients with apparent uterine confined disease. However, NCCN recommends para-aortic lymphadenectomy in high-risk patients including those with deeply invasive lesions, high grade histology, serous carcinoma, clear cell carcinoma, or carcinosarcoma (Category 2A recommendation). The result of pathologic assessment of lymph nodes may include: no lymph node metastasis, isolated tumor cells (ITC) (≤ 0.2 mm), micrometastases (> 0.2 – 2 mm), and macrometastases (> 2 mm). Micrometastasis and macrometastases are treated similarly as positive lymph node metastasis, while ITCs are considered node-negative. The basis for not treating ITCs includes retrospective data that found that subjects with ITCs had similar outcomes as subjects with negative lymph nodes and adjuvant treatment did not impact outcomes [8]. It is standard of care to treat patients with nodal metastasis with systemic therapy; NCCN guidelines specify systemic therapy (with or without radiation) as standard practice for surgically staged III and IV patients (Category 2A recommendation). This consensus is based on the high risk of recurrence in advanced stage patients, in one trial as high as 52% [9].

## Review of management options for front line treatment of node positive endometrial cancer

### Radiation only

Prior to effective systemic therapy, whole abdominal radiation was utilized in patients considered at risk for peritoneal failure. GOG 122 compared whole abdominal radiation to doxorubicin and cisplatin. Not only did chemotherapy treatment show improved progression free survival (PFS) and overall survival (OS), but whole abdomen abdominal irradiation had intolerable side effects and is appropriately no longer indicated [9–11].

### Radiation vs. Chemotherapy/Radiation (chemoRT)

PORTEC-3, a phase III randomized clinical trial (RCT), compared adjuvant chemotherapy during and after pelvic external beam radiation therapy (EBRT) versus EBRT alone in a high intermediate and high-risk EC cohort. Both arms received EBRT for a total dose of 48.6 Gy in 1.8 Gy per fraction 5 days a week. The chemoradiation arm also included two cycles of cisplatin 50 mg/m^2^ in the first and fourth weeks of EBRT followed by four cycles of carboplatin AUC5 and paclitaxel 175 mg/m^2^ at 21-day intervals. A total of 686 subjects with EC were included. There was an improvement in 5-year failure-free survival with the addition of chemotherapy from 68.6 to 75.5% (*P* = 0.02). The 5-year OS was 81.8% in the chemoradiation arm compared to 76.7% for RT alone which was not statistically different. However, post hoc analyses of stage III patients were noted to have improved OS (hazard ratio (HR) 0.63 [95% confidence interval (CI) 0.41–0.99]; *P* = 0.043) and failure free survival (FFS) (HR 0.61 [95% CI 0.42–0.89]; *P* = 0.011) [12•].

Important molecular subgroups and their relationship to survival were also evaluated in PORTEC-3 including p53 abnormal (p53abn), POLE ultra-mutated, mismatch repair-deficient (MMRd), and no specific molecular profile (NSMP). P53abn patients had the worst prognosis with 5-year recurrence free survival (RFS) of 48% and overall survival (OS) of 54%. The p53abn subjects in subgroup analysis had significant benefit from combined chemoRT compared to RT alone (5-year RFS 58.6% compared to 36.2%), suggesting substantial improvement with chemotherapy. POLE ultra-mutated subjects had the best outcomes regardless of adjuvant therapy, including 5-year RFS and OS of 98%. MMRd and NSMP had intermediate outcomes [12•, 13]. While this was a post-hoc analysis, it does provide a framework for differentiating molecular rather than histologic subtypes of EC that could particularly benefit from systemic therapy such as p53abn cases, and those who may be able to forego adjuvant therapy such as POLE ultra-mutated cases. Prospective trials designed to differentiate treatment by molecular subtype such as RAINBO [NCT05255653] for stage I-III disease, and PORTEC-4a [NCT03469674] (for early-stage high-intermediate disease) may provide confirmatory data to support this treatment paradigm (Table 1).

**Table 1:** Select ongoing trials that may change treatment of node positive endometrial cancer

NCT	Study Name	Phase	Summary of Inclusion Criteria	Treatment Groups	Primary Outcomes
NCT04567771	Comparison of acute toxicities between patients treated with protons or IMRT after surgery for EC or cervical caner	I	EC or cervical cancer treated with primary surgery. Planned to receive pelvic RT	Standard of care proton or IMRT therapy, and completion of quality of life questionaries and adverse event assessments	Change in Expanded Cancer Index Composite Bowel Score
NCT03660826	NRG- GY012	II	Recurrent or persistent EC. Requires one prior line of chemotherapy	Combination of olaparib and durvalumab, cediranib and durvalumab, olaparib and capivasertib, or cediranib alone	PFS
NCT05758688	PROPS GYN	II	EC or cervical cancer with indication for whole pelvic RT with or without systemic therapy	Adjuvant Whole Pelvis (WP) Pencil Beam Scanning Proton Radiation (PBS PRT). Doses of 45 or 50.4 Gy in 1.8 Gy daily fractions	Acute GI toxicity
NCT05255653	Refining Adjuvant Treatment IN Endometrial Cancer Based On Molecular Features (RAINBO)	II and III	-P53abn EC to Red Trial -MMRd EC to Green Trial -NSMP EC to Orange Trial -POLEmut EC to Blue Trial	-RED: adjuvant chemoRT followed by Olaparib vs. adjuvant chemoRT -Green: Adjuvant EBRT with and followed by durvalumab vs. adjuvant EBRT -Orange: Adjuvant EBRT followed by progestogens vs. adjuvant chemoRT -Blue: no adjuvant therapy for stage III and no adjuvant or EBRT for stage III	-Red: RFS -Green: RFS -Orange: RFS -Blue: Pelvic recurrence free survival
NCT05256225	NRG-GY026	II and III	HER2 positive, stage I-IV endometrial serous carcinoma or carcinosarcoma	Carboplatin/paclitaxel alone vs. carboplatin/paclitaxel combined with either trastuzumab and pertuzumab, or trastuzumab and hyaluronidase-zzxf	PFS, dose limiting toxicities, OS
NCT04634877	MK-3475-B21 / KEYNOTEB21 / ENGOTen11 / GOG- 3053	III	New diagnosis of EC including carcinosarcoma without history of radiation or systemic therapy	Pembrolizumab plus adjuvant chemotherapy vs. placebo plus adjuvant chemotherapy Either arm may receive RT	Disease free survival and OS
NCT05173987	MK-3475- C93/KEYNOT E-C93/GOG- 3064/ENGOTen15	III	EC including carcinosarcoma, inoperable stage IIIIV or recurrent. MMRd only	Pembrolizumab alone vs. carboplatin and paclitaxel for 6 cycles	PFS and OS
NCT05712941	GOG-3075	III	Grade 1 or 2 endometrioid EC, treatment naïve in advanced or metastatic setting	Letrozole and lerociclib vs. letrozole and placebo	PFS
NCT03469674	PORTEC-4a: Molecular Profile-based Versus Standard Adjuvant Radiotherapy in Endometrial Cancer	III	EC that is either: Stage IA, grade 3 (any age, ± LVSI) Stage IB, grade 1 or 2 age > 60 years Stage IB, grade 1 or 2 with LVSI Stage IB, grade 3 without LVSI Stage II (microscopic) Grade 1	Experimental molecular profile based treatment including vaginal brachytherapy, EBRT, or observation compared to standard treatment with vaginal brachytherapy	Vaginal recurrence

*EC* endometrial cancer, *p53abn* p53 abnormal, *MMRd* POLE ultra-mutated, mismatch repair-deficient, *NSMP* no specific molecular profile, *chemoRT* chemotherapy/radiation, *EBRT* external beam radiation therapy, *RT* radiation therapy, *RFS* recurrence free survival, *PFS* progression free survival, *OS* overall survival, *GI* gastrointestinal, *IMRT* intensity-modulated radiation therapy

### Combination cytotoxic ChemoRT vs. Chemotherapy only

GOG 177 and GOG 209 laid the foundation of carboplatin and paclitaxel combination therapy for high risk, advanced stage uterine cancer, as GOG 177 compared cisplatin and doxorubicin with or without paclitaxel in EC and GOG 209 demonstrated noninferiority of carboplatin and paclitaxel compared to cisplatin, doxorubicin, and paclitaxel [14, 15]. RTOG 9708 demonstrated anti-tumor activity of combination chemoradiation followed by chemotherapy [16]. The rationale of this regimen is based on synergistic effect of chemotherapy and radiation demonstrated in other tumors, as well as early receipt of a known effective systemic agent (platinum chemotherapy). GOG 258 asked a different question from PORTEC-3: Does chemoradiation improve survival compared to the standard chemotherapy alone? GOG 258 was a phase III RCT that included 813 subjects with stage III or IV EC. Approximately 75% of subjects in each arm had endometrioid stage IIIC1 or IIIC2 disease. This trial compared chemoRT (cisplatin 50 mg/m^2^ on days 1 and 29 in addition to volume-directed radiation at 45 Gy with or without brachytherapy, followed by carboplatin AUC 4 plus paclitaxel 175 mg/m^2^ every 21 days for four cycles) to chemotherapy alone (carboplatin AUC 6 plus paclitaxel 175 mg/m^2^ every 21 days for six cycles). Of note, 30% of patients received intensity-modulated radiation therapy (IMRT). Recurrence free survival was not improved with chemoradiation versus chemotherapy alone. Chemoradiation compared to chemotherapy alone did reduce the incidence of locoregional recurrence (2% vs 7%; HR 0.36) but was associated with more distant recurrences (27% vs 21%; HR 1.35). While there was a potential benefit in local–regional control with chemoRT, the actual proportion of patients impacted was relatively small, compared to the greater proportion of those afflicted with distant metastases which was likely driving the OS results. The most recent analysis with median follow-up time of 112 months revealed that chemoradiation did not improve OS over chemotherapy alone (HR 1.05, 95% CI 0.82–1.34), in any subgroup including histologic subtype [17, 18]. Similar to PORTEC3, molecularly based post-hoc analyses arecurrently ongoing. Chemotherapy alone remains standard of care for patients with advanced disease, but the addition of radiation may improve loco-regional control, and should be considered in patients with, for example, a positive margin or those at high risk for recurrence due to extensive local disease (e.g. parametrial or vaginal involvement).

## Targeted therapy, a new frontier in node positive endometrial cancer treatment

The emerging direction of cancer research and treatment is targeted therapy, which the National Cancer Institute defines as a treatment that uses drugs or other substances to target specific molecules in order to prevent the survival and spread of cancer cells. Examples of targeted therapy include drugs that inhibit growth of blood vessels, improve function of the immune system, or utilize antibodies to deliver small-molecule drugs.

### Immunotherapy

Pembrolizumab is an anti-PD-1 humanized monoclonal antibody that is utilized as an immune checkpoint inhibitor. Keynote-158 was a nonrandomized, open-label phase II trial which explored the efficacy and safety of Pembrolizumab alone in MMRd or microsatellite instability high (MSI-H) previously treated, advanced EC. Objective response rate was high (48%) with median duration of response not yet reached at 42.6 months. This study leads to the accelerated approval of pembrolizumab in previously treated advanced or recurrent endometrial carcinoma [19]. Dostarlimab is also an anti-PD-1 monoclonal humanized antibody utilized in EC. The Garnet trial was a single group open label clinical trial assessing dostarlimab’s impact on survival in MMRd and mismatch repair proficient (MMRp) EC subjects. Stage III patients comprised 35% of the MMRd cohort and 28% of MRRp cohort. The overall response rate (ORR) was 43.5% (95% CI 34.0 – 53.4) in MMRd patients and 14.1% (95% CI 0.1 – 20.6) in MMRp patients. The median duration of response was not reached in either cohort. [20] These results suggest that MMRd patients may have a particularly high response rate to single agent immunotherapy, and that MMRp may have a modest response rate, but potentially on par historically with other single cytotoxic agents.

These trials shifted the paradigm of treatment. Recently two phase III, double blind, placebo-controlled, RCTs combining immunotherapy and chemotherapy followed by immunotherapy maintenance in EC have been published: RUBY and NRG-GY018. Both trials included node-positive EC subjects. The results of these trials have already been incorporated into NCCN guidelines for advanced endometrial cancer.

Both RUBY and GY018 were presented at the Society for Gynecologic Oncology 2023 Annual Meeting and published in the New England Journal of Medicine the same day.  GY018 utilized pembrolizumab while Ruby utilized dostarlimab. A summary of similarities and differences of these trials is listed in Table 2 for reference [21••, 22••].  In both trials the improvement in PFS for the MMRd population was impressive. In RUBY progression-free survival at 24 months was 61.4% (95% CI, 46.3 – 73.4) in the dostarlimab group and 15.7% (95% CI, 7.2 – 27.0) in the placebo group (HR for progression or death, 0.28; 95% CI, 0.16 – 0.50; *P* <0.001).  In GY018, MMRd subjects on pembrolizumab had improved PFS at 12 months:  Kaplan–Meier estimates of freedom from disease progression or death were 74% v 38%, (HR 0.30; 95% CI, 0.19 -0.48; *P *< 0.001). In GY018, the MMRp population showed less clinical benefit but was still statistically significant; median PFS 13.1 months with pembrolizumab vs 8.7 months with placebo (HR 0.54; 95% CI, 0.41 – 0.71; *P *< 0.001). In RUBY, the MMRp subjects PFS at 24 months was 28.4% (95% CI, 21.2 – 36.0) in the dostarlimab group and 18.8% (95% CI, 12.8 – 25.7) in the placebo group (HR for disease progression or death, 0.76; 95% CI, 0.59 – 0.98).

**Table 2:** Summary of RUBY and NRG-GY018 clinical trials

Study	RUBY	NRG-GY018
Treatment	Randomized 1:1 to carboplatin AUC 5, paclitaxel 175 mg/m2, and dostarlimab 500 mg q3w for 6 cycles followed by dostarlimab 1000 mg q6w up to 3 years vs. placebo q3w for 6 cycles followed by placebo maintenance	Randomized 1:1 to carboplatin AUC 5, paclitaxel 175 mg/m2 and pembrolizumab 200 mg q3w for 6 cycles followed by pembrolizumab 400 mg q6w up to 14 cycles vs. placebo q3w for 6 cycles followed by placebo maintenance
Inclusion criteria	-Primary endometrioid EC stage IIIA, IIIB, or IIIC1 with measurable disease -Primary endometrioid EC stage IIIC2 or IV regardless of measurable disease -Primary carcinosarcoma, clear-cell, serous, or mixed histologic characteristics, stage IIIC1 + regardless of measurable disease -First recurrence at least 6 months after completion of systemic chemotherapy -May have had prior EBRT	-Primary EC Stage III—IVA with measurable disease -Primary EC Stage IVB disease with or without measurable disease -Recurrent EC with or without measurable disease -Included all histologies except carcinosarcoma -Recurrence at least 12 months after completion of systemic therapy -May have received prior pelvic radiation (EBRT/brachytherapy) a least 4 weeks prior to step 2 registration
Enrollment	494 subjects were randomized from July 2019 – February 2021	816 subjects were randomized from July 2019—December 2022
Primary Endpoints	PFS for MMRd, MSI high subjects and overall population, and OS for overall population	PFS for MMRd and MMRp subjects
Median Follow up	25.4 Months of follow up in overall population	12 months in MMRd population, 7.9 months in MMRp population
Results	PFS at 24 months: -MMRd: 61% with dostarlimab vs. 15.7% with placebo. Median PFS not reached with dostarlimab vs. 7.7 months with placebo -MMRp: 28% with dostarlimab vs. 18.8% with placebo. Median PFS with dostarlimab 9.9 months vs. 7.9 months with placebo	PFS at 12 months (MMRd), 7.9 months (MMRp): -MMRd: 74% with pembrolizumab vs. 38% with placebo. Median PFS not reached with pembrolizumab vs. 7.6 months with placebo -MMRp: 46% lower risk with pembrolizumab. Median PFS 13.1 months with pembrolizumab vs. 8.7 months with placebo

*EC* endometrial cancer, *MMRd* mismatch repair-deficient, *MMRp* mismatch repair-proficient, *EBRT* external beam radiation therapy, *MSI* microsatellite instability, *PFS* progression free survival

Based on the findings of these studies, the NCCN guidelines have been updated to include combination therapy with pembrolizumab/carboplatin/paclitaxel and dostarlimab/carboplatin/paclitaxel as Category 1, preferred, primary therapy options for stage III or IV endometrial carcinoma. July 31, 2023 the FDA approved dostarlimab with carboplatin and paclitaxel followed by single agent dostarlimab for primary or recurrent MMRd EC. Specific recommendations for treatment with measurable disease or less common histologies are based on the inclusion criteria of each study [21••, 22••]. For example, carcinosarcoma was included in RUBY but excluded from GY018. GY018 required measurable disease for all patients except recurrent patients.  RUBY required measurable disease for endometrioid IIIC1 patients, however, did not require measurable disease for carcinosarcoma, serous, clear cell, or mixed histologies, nor for IIIC2 or IV cases. GY018 enrolled patients who recurred after 12 months, whereas RUBY enrolled patients who recurred after 6 months. Given that neither GY018 nor RUBY enrolled IIIC1 endometrioid patients with no residual disease, it remains unclear whether this subset of patients would benefit from combined cytotoxic chemotherapy plus immunotherapy. This question may be answered by Keynote B21/GOG 3053 trial which is enrolling all stage III patients with any uterine cancer histology and randomizing to pembrolizumab with carboplatin/paclitaxel followed by pembrolizumab maintenance versus placebo with carboplatin/paclitaxel followed by placebo maintenance (Table 1). In this trial, the decision to perform radiation after chemotherapy is institution dependent, but radiation continues to be permitted. The results of GOG 3053 will also be stratified by MMR status.

Adverse events (AEs) are a limitation of combination therapy, both for chemoRT and chemo-immunotherapy. Approximately 60% of PORTEC-3 subjects had grade 3 + AEs in the chemoRT arm compared to 12% in RT alone. The most significant AE in the chemoRT arm was neuropathy [12•]. In GOG 258, about 58% of subjects in the chemoRT arm experienced Grade 3 + AEs compared to 63% for chemotherapy alone. Hematologic AEs were more frequent and severe in the chemotherapy arm [23]. In RUBY, nausea, alopecia, and fatigue were high (about 50%) but similar between groups. Rash was the most notable AE experienced in the treatment arm. Grade 3 + events were about 10% higher in the treatment group [21••]. In GY018, the grade 3 + AEs were divided by MMR status. In MMRd subjects, 63% on pembrolizumab experienced a Grade 3 + AE compared to 47% on placebo. In MMRp subjects, 55% experienced a Grade 3 + AE on treatment compared to 45% on placebo. Adverse events related to immunotherapy in the treatment arms included infusion reaction (15%), hypothyroidism (13%), hyperthyroidism (9%) [22••].

Several important questions remain regarding immunotherapy for node positive endometrial cancer patients:

- Can advanced MMRd endometrial cancer patients be treated with immunotherapy alone? The MK-3475-C93/KEYNOTE-C93/GOG-3064/ENGOT-en15 study is an actively recruiting phase III, randomized, open label clinical trial comparing Pembrolizumab to platinum doublet chemotherapy in MMRd advanced or recurrent EC (Table 1).
- Do we treat MMRp stage IIIC1 patients with no measurable disease with combined immunotherapy and chemotherapy given these patients were not included in RUBY or GY018 at baseline and the clinical benefit for MMRp patients was significantly less? GOG 3053 will shed light on this question, as well as whether combined immunotherapy and EBRT is tolerable and effective for Stage IIIC1 patients (Table 1).
- For stage IIIC2 patients, who showed clinical benefit from addition of pembrolizumab or dostarlimab plus immunotherapy maintenance, how do we counsel them regarding radiation therapy? Are there any additional safety considerations?
- How do we incorporate molecular classification into our treatment protocols? Do we escalate or de-escalate treatment at this time based on molecular classification? The RAINBO trial (enrolling stage I-III) and PORTEC 4 (enrolling stage I-II) is stratifying patients into p53abn, MMRd, NSMP, and POLEmut groups for randomization and treatment (Table 1).

### HER2/ERBB2

Human epidermal growth factor receptor 2 (HER2)/neu functions in cell growth, survival, and proliferation, and is overexpressed in 4–69% of endometrial carcinoma [24, 25]. Trastuzumab is a monoclonal antibody that targets HER2.

A Phase II randomized clinical trial comparing carboplatin/paclitaxel for six cycles versus carboplatin/paclitaxel/trastuzumab followed by trastuzumab maintenance in patients with stage III/IV or recurrent, HER2/neu positive uterine serous carcinoma showed clinical benefit from the addition of trastuzumab. HER2/neu positivity was defined as having an immunohistochemical (IHC) score of 3 + or 2 + *and* amplification on fluorescence in situ hybridization (FISH). PFS was significantly improved with trastuzumab (12.6 months in the experimental arm versus 8.0 months in the control arm, HR 0.44; 90% CI, 0.26 to 0.76, *P* = 0.005). Secondary outcome of OS did not reach statistical significance, though hazard ratio showed mortality reduction in the trastuzumab arm (HR 0.41) [26•]. The greatest benefit appeared in patients receiving frontline therapy for stage III–IV (HR, 0.40; 90% CI, 0.20 to 0.80, *P* = 0.013) rather than in the recurrent setting (HR, 0.14; 90% CI, 0.04 to 0.53, *P* = 0.003). NCCN guidelines include the addition of trastuzumab for stage III/IV HER2 + uterine serous carcinomas or carcinosarcomas (Category 2A recommendation).

A follow-up phase III study is currently recruiting NRG-GY026, a randomized phase II/III trial comparing trastuzumab and pertuzumab versus trastuzumab and paclitaxel/carboplatin in patients with HER2 positive endometrial serous carcinoma and carcinosarcoma. This trial includes chemo-naïve, non-recurrent stage IA–IVB uterine serous or carcinosarcoma that is HER2 + (Table 1).

### Hormonal therapy as monotherapy or in combination

Approximately 85% of endometrial cancers are found to be hormone receptor positive on IHC analysis [27]. NCCN guidelines consider hormonal therapy as a Category 2A treatment option for recurrent or metastatic disease. GOG 3007 was a single stage, open label randomized phase II trial for advanced or recurrent EC treated with everolimus and letrozole (EL) or medroxyprogesterone acetate and tamoxifen (MT). Median PFS was 6 months (95% CI 3.8 – 17.7) in EL versus 4 months (95% CI 2.7 – 6.1) in MT; higher PFS was observed in chemo-naïve patients [23]. While cytotoxic chemotherapy was associated with longer PFS improvement, hormonal therapy may be a good option for patients who desire a break from cytotoxic agents or cannot tolerate chemotherapy.

An actively recruiting clinical trial studying hormonal therapy in advanced EC is GOG-3075. This is a randomized, double-blind placebo-controlled phase III trial comparing lerociclib (selective CDK4/6 inhibitor) with letrozole to placebo with letrozole in patients with Grade 1/2 endometrioid endometrial cancer with advanced or metastatic disease (Table 1).

### PARP inhibitor

There are currently no approved poly (ADP-ribose) polymerase (PARP) inhibitors for the treatment of EC. Musacchio et al. summarized the in vitro and in vivo studies of PARP inhibitors and EC. As p53 and PTEN mutations are common in EC and associated with the homologous recombination pathway, PARP inhibitors may have a role to play in treatment [28]. There are multiple clinical trials looking into whether PARP inhibitors show clinical benefit including NRG- GY012, a randomized phase II study comparing single-agent olaparib, single-agent cediranib, and combination olaparib/cediranib in women with recurrent, persistent, or metastatic endometrial cancer (Table 1). The RAINBO trial also is utilizing PARP inhibitors in a maintenance fashion in p53abn EC.

## New technology in radiation oncology

While the role of RT will continue to be personalized in the context of disease risk and other systemic therapies, significant improvements in RT have led to clinically significant reductions in toxicity while maintaining disease control. Modern EBRT is delivered nearly exclusively using IMRT technique. Most adjuvant radiation studies for uterine cancer have used 2D (e.g., in PORTEC 1 and 2) or 3D external beam radiation (also known as 3D conformal radiation) techniques which indiscriminately radiates bowel and bone marrow, particularly if extended field radiation is used (for example, to treat the para-aortic nodes). IMRT uses multiple fields to shape the high dose radiation treatment to, better spare bowel, bone marrow, bladder, and rectum potentially mitigating risks of nausea, enteritis, and cystitis.

The RTOG 1203/TIME-C trial randomized women with post-operative uterine or cervix cancer to 3D conformal radiation vs IMRT to 45–50.4 Gy with or without concurrent platinum therapy. The use of IMRT compared to 3D led to significantly improved patient reported quality of life (QOL) in genitourinary and gastrointestinal toxicities [29•]. IMRT is now the recommended treatment for all patients in the post-operative setting, including in ongoing clinical trials. When counseling patients, IMRT results in fewer GI and GU toxicities, and toxicities with extended field radiation to treat PA nodes are significantly mitigated with IMRT. While not in uterine cancer, the PARCER trial for post-operative treatment of cervix cancer confirmed smaller reductions in quality of life with comparable disease control in IMRT compared to 3D radiation [30]. Particle therapy using protons may further improve clinical outcomes by further reducing dose to bowel and bone marrow [31, 32]. The APROVE Phase II study from Heidelberg benchmarked clinical outcomes for proton therapy for treatment of the whole pelvis [33], and clinical trials in the USA are underway (NCT05758688, NCT04567771) (Table 1). IMRT for post-operative external beam treatment of uterine cancer is firmly standard of care, and further refinements in technique may reduce toxicity even more and maintain in-field control.

Given the use of external beam radiation may decrease in initial adjuvant management of node positive uterine cancer, we may expect to see a rise in use in nodal and distant recurrences. In patients with measurable pelvis-only nodal disease, both radiation therapy alone per GOG238 or systemic chemoimmunotherapy can be standard of care. However, the latter regimen is likely not curative on its own. In patients with low-volume or oligometastatic lesions, there may be an enhanced role for the use of stereotactic body radiation therapy (SBRT), also known as stereotactic ablative body radiation (SAbR). SBRT combines the conformality of IMRT with robust immobilization methods to ablate lesions with millimeter accuracy inside the body. This may allow ablation of metastatic lesions or retreatment of measurable disease in previously radiated fields safely [34]. SBRT combined with best systemic therapy for measurable disease recurrence may allow for optimal treatment of recurrent disease or delaying of initiation of systemic therapy.

The incorporation of adjuvant external beam radiation is generally not performed in high-risk histologies with node-positive disease in GOG studies utilizing chemotherapy given the very high risk of distant recurrence in these patients. For example, all uterine carcinosarcoma were excluded from GOG 258. Again, while the use of radiation for endometrioid subtypes may decrease, there may be an unexpected reinvigoration in its importance particularly in patients who are less likely to respond to optimal systemic therapy. For example, patients with Grade 1 endometrioid adenocarcinoma, NSMP, or MMRp subtypes may respond less often to best systemic therapy, or may be better tailored with immunotherapy, hormonal, or targeted therapy with potentially less toxicity, and the re-addition of external beam radiation may improve outcomes in these patients [35].

## Conclusion

The management of node positive EC is changing in the setting of refined molecular classification, new RT modalities, and targeted therapy. While clinicians await the results of ongoing clinical trials regarding combined immunotherapy/RT as well as management based on molecular classification, we must make decisions regarding the best treatment combinations for our patients. Based on the available literature, we are offering stage IIIC endometrioid EC patients without measurable disease postoperatively both IMRT and chemotherapy with carboplatin, paclitaxel, and with or without pembrolizumab/dostarlimab as primary adjuvant therapy. In patients who desire both chemoimmunotherapy and radiation, we are treating patients with six cycles of chemoimmunotherapy followed by IMRT. Patients with measurable disease post operatively, all high risk histologies, or stage IV disease are receiving chemoimmunotherapy; those with uterine risk factors for vaginal recurrence such as deep muscle invasion, lymphovascular invasion, and/or high risk histology are additionally given vaginal brachytherapy. For patients receiving immunotherapy maintenance, duration of treatment is in accordance with published literature (14 cycles with pembrolizumab and up to 3 years with dostarlimab) [21••, 22••]. In patients receiving both radiation and immunotherapy, we continue immunotherapy during radiation treatment. We are also applying molecular testing, particularly using p53abn and MMRd to guide our adjuvant therapy recommendations as well as prognosis discussions rather than histology alone. For example, in patients with p53abn tumors, we are prioritizing systemic therapy, and in the MMRd population we prioritize chemoimmunotherapy when appropriate. As for POLEmut cases, we are not currently de-escalating adjuvant treatment recommendations, but are using that information in our counseling to discuss expected efficacy and outcomes. In the setting of endometrioid EC recurrence more than 6 months after treatment, patients with vaginal recurrence are offered IMRT and brachytherapy without chemotherapy. For measurable recurrence not suitable for pelvic radiation alone, chemoimmunotherapy is preferred as standard of care.
